# Reconsidering the Relationship between Air Pollution and Deprivation

**DOI:** 10.3390/ijerph15040629

**Published:** 2018-03-29

**Authors:** Nick Bailey, Guanpeng Dong, Jon Minton, Gwilym Pryce

**Affiliations:** 1 School of Social and Political Sciences, University of Glasgow, Glasgow G12 8QQ, UK; Nick.Bailey@glasgow.ac.uk (N.B.); nate.minton@gmail.com (J.M.); 2 Department of Geography and Planning, University of Liverpool, Liverpool L69 7ZT, UK; 3 Sheffield Methods Institute, Faculty of Social Sciences, University of Sheffield, Sheffield S1 4DP, UK; g.pryce@sheffield.ac.uk

**Keywords:** air pollution, geographically weighted regression, spatiotemporal variations, deprivation

## Abstract

This paper critically examines the relationship between air pollution and deprivation. We argue that focusing on a particular economic or social model of urban development might lead one to erroneously expect all cities to converge towards a particular universal norm. A naive market sorting model, for example, would predict that poor households will eventually be sorted into high pollution areas, leading to a positive relationship between air pollution and deprivation. If, however, one considers a wider set of theoretical perspectives, the anticipated relationship between air pollution and deprivation becomes more complex and idiosyncratic. Specifically, we argue the relationship between pollution and deprivation can only be made sense of by considering processes of risk perception, path dependency, gentrification and urbanization. Rather than expecting all areas to eventually converge to some universal norm, we should expect the differences in the relationship between air pollution and deprivation across localities to persist. Mindful of these insights, we propose an approach to modeling which does not impose a geographically fixed relationship. Results for Scotland reveal substantial variations in the observed relationships over space and time, supporting our argument.

## 1. Introduction

Revelations in 2015 that car manufacturers had been evading environmental regulations focused public attention on the issue of air pollution and the harm caused by diesel vehicles in particular [1]. Since then, a major review by the Royal College of Physicians (RCP) has further highlighted the importance of the problem, concluding that air pollution has led to 40,000 premature deaths annually in the UK [2]. Impacts on this scale raise important questions about distributional impacts and the environmental justice (EJ) of air pollution. We focus in this paper on the relationship between air pollution and deprivation, which are often assumed or presented as having a positive association. For example, data depicted in the RCP review [2] (p. 73) appear to show a simple positive, linear association between the area deprivation and exposure to various adverse environmental conditions, including poor air quality. 

It would be implausible to suggest a direct causal relationship between pollution and deprivation: that more deprived households are responsible for higher pollution. Why might we expect the correlation between deprivation and pollution to be positive? There are two main reasons. First, low income households have fewer economic resources with which to compete for better quality neighborhood amenities so the market sorts them towards locations with lower quality, and we might expect that to include lower air quality. Second, planning processes may act to reinforce this distribution by siting undesirable amenities, including those generating air pollution, closer to lower income areas—the central concern of the US-led environmental justice (EJ) literature from the 1970s [3]. Even when planners distribute environmental disamenities more equitably, market sorting may lead to poorer households “coming to the nuisance” as house prices fall in neighborhoods close to new disamenities [4]. 

Since the underlying processes noted here are universal ones, we would expect to see a similar pattern everywhere and we would expect any deviations from this state to be temporary. The implication is that the environmental injustice of air pollution exposure is both persistent and potentially intractable. The situation is made all the more objectionable because those that bear the greatest burden of pollution are the least involved in generating it. For example, a large proportion of air pollution is generated by car use, but cars, being costly to own and to run, are disproportionately driven by richer households [5]. 

With rapid increases in car use in many countries from the 1950s onwards, and better understanding of the health risks of airborne pollutants, there has understandably been growing interest in the EJ research regarding the relationship between area deprivation and the spatial variation in air pollution [6,7,8,9,10]. However, although the findings of existing studies generally suggest a positive correlation, there have also been some indications in the literature that association between air pollution and deprivation might be more complex than that described by a simple linear relation. For example, with respect to the distribution of pollutants in the UK, the RCP report notes that “the relationship with deprivation is not straightforward” [2] (p. 73) and [5] found that, at the time of the 2001 census, the relationship between deprivation and air pollution was J-shaped. That is, both the most affluent areas and some of the most deprived areas experienced higher levels of air pollution, and areas of middling deprivation were exposed to least pollution on average. 

In this paper, we aim to explore in more detail why the relationship between pollution and deprivation might be a complex one: potentially non-linear, negative or non-monotonic. We argue that EJ research should adopt a more pluralistic theoretical perspective derived from a broader range of urban theory, urban economics and behavioral economics approaches [4,11,12]. Specifically, we argue the relationship can only be made sense of by considering processes of risk perception, gentrification and urbanization, and by thinking more carefully about how increased spatial population density—i.e., moving to a city—tends to bring both benefits (agglomeration effects) and harms (congestion effects). 

Focus on a particular economic or social model of urban development can lead one to expect all cities to be converging towards a particular universal norm. A simple market sorting model, for example, might lead one to expect poor households to eventually be sorted into high pollution areas. If, however, one considers a wider set of theoretical perspectives, one might expect the relationship air pollution and deprivation to be rather more idiosyncratic. Not only might it not be universally positive and monotonic, but the relationship may also be spatially and temporally varying and persistently so due to path dependencies: local historical differences in development and topology lead to long term differences. Meanwhile, short term variations in demography and economic activity can have non-trivial impacts on the location of pollution and poverty, leading to fluidity over time in the relationship. Both spatial variations and temporal fluidity should therefore be viewed as potentially persistent features, rather than temporary states on the road to convergence to a uniform long-run equilibrium. Hence, the aims of this paper are first to encourage a broader theoretical outlook which supports the idea of spatially and temporally varying relationships; and second to demonstrate an approach to empirical modeling which captures such variations. We apply this approach to data for Scotland, showing substantial spatiotemporal variability in the observed pollution–deprivation relationship.

The structure of the rest of the paper is as follows. In the next section, we outline in more detail the various theoretical arguments which might lead to more complex relationships between air pollution and deprivation. We then outline our proposed method, and summarize the data used in the analysis. Finally, we present our findings and conclude with a brief summary, offering some tentative thoughts on the implications for policy and suggested avenues for future research. 

### A More Pluralistic Theoretical Framework

A range of theoretical perspectives lead us to question whether the relationship between environmental quality and deprivation will necessarily be positive, and to suggest that the relationship may vary over space and time in a persistent fashion. Different causal processes generating the relationship, with a focus on the discriminatory siting of environmental disamenities and post-siting population dynamics in the general environmental justice literature, have been reviewed in [13]. The theoretical grounds on the spatiotemporal nonlinearity of the relationship is however not explicitly discussed and tested empirically, which is the focus of the study.

Why Not a Positive Relationship? 

Two arguments can be mobilized to challenge the expectation of a positive relationship between air pollution and deprivation: the existence of location trade-offs, combined with heterogeneity of preferences; and lack of awareness of air pollution or understanding of its impacts. 

Location Trade-Offs and Heterogeneous Preferences

Neighborhoods are composite commodities or “bundles of goods”, some private and some public, some desirable and some not, and some more attractive to some types of household than others [12,14]. There is an extensive literature using hedonic models to estimate the value of individual neighborhood characteristics such as proximity to a train station or a park [15,16,17,18,19]. It is also clear that many neighborhood characteristics are clustered together in complex ways, with some generally seen as “bad” often positively associated with others seen as “good” neighborhood characteristics [20,21]. Households are therefore faced with complex trade-offs. 

This paper is focused on one such association: that which exists between agglomeration economies and air pollution. Both arise broadly from density, although the basis for the relationship has altered as cities have undergone the shift from industrial to post-industrial economies. As industrial centers, the employment locations within cities were simultaneously the attractors for households and the generators of a substantial proportion of air pollution [22,23]. With relatively low levels of mobility, commuting distances were short so richer and poorer households lived close to these locations, adding to air pollution through domestic combustion of polluting fuels such as coal. With the shift to a post-industrial economic base in cities across the developed world, urban economic activities are no longer so locally polluting while domestic energy consumption produces far less pollution in cities, partly because of the switch to cleaner fuels, and partly because households have spread to suburban and ex-urban locations [24,25,26,27,28]. Instead, the urban economy now produces a larger proportion of its damaging effects on air quality through commuting and other travel flows, particularly those made by the same cars which enabled suburbanization [5]. 

Ongoing changes to urban structures, combined with complex interactions with local social and cultural trends and path dependencies, suggest a potentially fluid and complex relationship between air pollution and deprivation over time. For example, rising car ownership may have enabled more affluent groups in particular to remove themselves from the most polluted locations, but it is clear that not all have chosen to do so. Preferences are more heterogeneous. Indeed, various “Back to the City” counter-movements are led by some of the most affluent groups [29,30,31,32].

The processes of de-industrialization have created the opportunity spaces for gentrification in former industrial buildings and dwellings that would previously have housed industrial workers, as well as creating the social groups that drive gentrification, notably the younger, better-educated workers of the post-industrial economy. In many cities, a culture of urban living has been created, rediscovered or imported which is attractive at least to some with high levels of locational choice in the housing system. For these households, the opportunities afforded by living in dense urban locations are more than offset by the downsides, including air pollution. 

Lack of Awareness or Understanding

The first point assumes that individuals are both aware of levels of air pollution in their locality and motivated by the risks which these pose. There is however an extensive literature on environmental risk perceptions which questions both assumptions (see Bickerstaff 2004 for a detailed review) and which leads us to further question whether there is likely to be a simple, stable positive relationship between air pollution and deprivation. First, there are psychological and behavioral economics literature which suggests we are poor judges of air pollution and similar environmental risks [11]. We exhibit perceptual biases, notably the tendency to discount environmental risks in our own neighborhood (the “neighborhood halo” effect). Even when we identify risks, we tend to play down the potential for adverse effects to impact on ourselves (“personal invulnerability”). Furthermore, our perceptions of air quality are strongly influenced by visual stimuli (soot or dirt, or pollution “haze”, for example) even though these provide only a partial guide to the aspects of air quality which have most influence on health. 

Second, there is a growing contribution from disciplines such as geography and sociology on the social and cultural influences on our perceptions. A place which had pollution in the past may retain a reputation or stigma which bears little relation to current risks. Conversely, we might expect positive reputations to last even once air quality has deteriorated. Looking at social differences, more powerful groups with a greater sense of personal or collective agency (crudely, white, male, or more affluent, for example) are more likely to discount risks and to demonstrate a greater sense of personal invulnerability. Thus, although these groups may have greater opportunity to avoid more polluted locations by virtue of their economic resources, they may be less motivated to do so. The “neighborhood halo” is also stronger for those who feel positively about their neighborhood, which is more likely to be the case for the more affluent groups in less deprived locations [33]. Moreover, the process of updating our knowledge and the transition to more informed understandings of environmental risk are not necessarily gradual or convergent over time, but characterized variously by periods of inertia, myopia, sudden realization, and amnesia [11]. The complexity and fluidity implied by our bounded rationality and knowledge acquisition implies similar potential characteristics for the relationship between deprivation and pollution. 

Why Spatial Variation, Persistent Divergence and Fluidity?

We can also identify two sets of arguments for why the relationship between pollution and deprivation may vary over space in a stable fashion, as well as going through periodic shifts which cannot be described as simple convergence. These relate to issues of path dependency and the de/re-commodification of housing stocks.

Path Dependency

Cities and city regions are complex, historically contingent systems [34,35]. Physical development is relatively durable, and past forms and uses constrain current development possibilities. Cities and regions each developed in different physical environments, under different social and economic conditions, and adapted in response to different challenges and opportunities. This means that a common schedule of congestion and agglomeration effects as a function of the density of human activity, and the trade-offs or pay-offs that come with density, cannot be assumed to apply equally well, or to be at the same stage of evolution, for all city regions. 

In addition, there is strong path dependence in neighborhood hierarchies. Reference [36] shows how the reputation of a neighborhood, established when it is first constructed, may influence its status many decades later. Reference [37] shows that deprived neighborhoods may find it difficult to shake off their stigmatized status even after substantial investment and social change. Localized externalities are another source of persistence. Positive amenities such as high quality public or private services serve to attract more affluent groups which, in turn, sustain those services either through their purchasing power or through active interventions in political processes—the “sharp-elbowed” middle classes [38]. Neighborhoods can and do change but such path dependence means change may be slower or more discontinuous rather than a process of smooth adjustment. In other words, significant changes in levels of air pollution may not generate corresponding changes in residential status or mix, or may do so in different ways and at different speeds in different cities. 

De-Commodification of Housing

In general, the argument from sorting models is that even altruistic planning decisions are ineffectual in re-distributing environmental disamenity in the longer term as market sorting serves to re-establish social gradients in parallel with disamenity [4]. One significant exception here may be where housing is de-commodified so that market sorting processes no longer apply. In theory, this is the case with much social housing, since access is via bureaucratic allocations based on needs assessment, not economic resources [39]. The consequences for the pollution–deprivation relationship will depend on the extent of de-commodification and the spatial distribution of the social housing stock, and will therefore vary between cities and over time. Scotland, the focus of the empirical material later in this paper, has a relatively large social rented sector which housed more than half the population at its peak at the start of the 1980s and which continues to be home to almost one in four. In England, by contrast, it never reached one in three, and has now fallen to one in six. Within Scotland, levels of social housing were much higher in urban than rural areas, and this may reinforce the link between deprivation and pollution. Within the cities, however, concentrations of social housing are found both in the urban core on the sites where slum clearance occurred and in large developments at the periphery where undeveloped land was available at the time. 

Not only does the level of social housing vary between locations and over time, so too does the extent to which it is socially selective or targeted only on more deprived groups—compare, for example, the dual rental market system characteristic of Anglo-Saxon countries [40] with unified rental systems which tend to be characterized by a much more diverse social housing population. Even within the one system, the degree of selectivity may vary over time depending on allocations policies [39]. Policy changes, such as “Right to Buy”, and the transfer of the social housing stock from municipal ownership to housing associations, can cause major shifts in the patterns of housing tenure, adding to the complexity and temporal discontinuity in the pollution–deprivation relationship.

Summary

These various theoretical processes have the potential to generate changes over time and across space in the relationship between pollution and deprivation. The potential for interaction between these processes, including location trade-offs, heterogeneous preferences, bounded rationality, path dependencies and housing governance, will only exacerbate the potential complexity of outcomes. The corollary is that we should not assume that the relationship between deprivation and pollution will be linear, positive, monotonic or fixed in time or space. This presents us with an imperative to deploy empirical methodologies that are sufficiently flexible to cater for these features. It is these which we turn to now. 

## 2. Materials and Methods 

### 2.1. Analytical Approach

We sought an analytical approach that would enable us to identify both non-linear and spatially-varying relationships between pollution and deprivation. In the first stage, we explore the relationships without imposing functional form, plotting median pollution by deciles of deprivation for Scotland and for the four main city-regions to examine whether there is initial evidence of spatial or temporal variation in the relationships. Here, we impose a definition of regions or sub-areas (a “top down” approach) rather than allowing them to emerge from the data. We summarize the relationships using simple linear models for Scotland and for each of the city-regions:

(1) $$pollution_{i}=\;\beta_{0}+\;x_{i}^{T}\gamma +\;\varepsilon_{i};\;i\;=\;1,\;2,\;\ldots ,\;N$$

In Equation (1), *pollution_i_* is air pollution concentration for Datazone *i* (e.g., PM_2.5_) while *x_i_^T^* contains linear and quadratic terms of Income deprivation at Datazone *i* (centered on the annual mean) and *γ* is a 2 by 1 vector of regression coefficients, assumed constant across the region; higher-order polynomials were tested but did not add sufficiently to justify the additional complexity. *β*_0_ is the intercept, and *ε_i_* residuals assumed to follow a normal distribution. 

In the second stage, we explore spatial variations in the pollution–deprivation relationship without imposing geographic boundaries using a Geographically Weighted Regression (GWR) model, which allows the relationships between variables to vary across a study region [41]. GWR is a localized model fitting process where a sequence of regression models is estimated across the study area, each centered on one fit point (Datazone). In each model, weights are inversely proportional to the geographic distance between the fit point and the observation, giving most weight to observations closest to the fit point, reducing to zero at some threshold distance. After fitting the models, regression coefficients for each explanatory variable can be compared while statistical tests can identify whether there is significant heterogeneity in the covariate effect. Extending Equation (1), the models can be written:

(2) $$pollution_{i}=\;\beta_{0i}+\;x_{i}^{T}\gamma_{i}+\;\varepsilon_{i};\;i\;=\;1,\;2,\;\ldots ,\;N$$

To estimate the GWR models, a weighting scheme and a bandwidth parameter need to be chosen. Commonly employed weighting schemes include Gaussian, exponential or bi-square kernel function [41]. A further decision is choosing between a fixed GWR (in which the bandwidth relates to a fixed physical distance) and an adaptive GWR (in which bandwidth is defined as the n nearest neighbors). In the latter, the kernel adapts to the local density of samples, extending over a larger physical area where samples are more sparsely distributed [42]. Given large variations in residential density across Scotland, we use the adaptive approach based on the 400 nearest neighbors. See the Supplementary Materials for more information on the choice of models. In addition to presenting results in tabular form, they are also mapped so that the spatial patterning is more apparent.

The final stage uses the results of the GWR to identify regions where the pollution–deprivation relationship is similar (a “bottom up” approach, in contrast to the first stage). Cluster analysis is employed to group Datazones with similar parameter values [43]. We use the standard k-means algorithm to conduct the cluster analysis of the spatially-varying coefficients.

### 2.2. Data

To demonstrate the proposed analytical approach of environmental injustice research that highlights non-linearity, spatially varying relationships and temporal dynamics, we investigate the associations between pollution concentration and deprivation at the national and city-region scales. The spatial units used in the analysis are Datazones, which are designed to be relatively homogenous areas with similar population sizes [44]. Using the original Datazones built from the 2001 Census and used up to about 2012, there are 6505 Datazones for Scotland as a whole with population counts usually ranging between 500 and 1000.

For each Datazone, the Scottish government produces a measure of Income deprivation as part of the official index of small area deprivation, the Scottish Index of Multiple Deprivation (SIMD). This is available for the years 2004, 2006, 2009 and 2012 so these are the basis for our analysis. The SIMD is built from many indicators, selected to capture deprivation on several separate domains, and combined to give an overall deprivation score. The selection of indicators their relative weights both change over time making comparisons problematic. The Income deprivation domain captures the proportion of the population in receipt of a low-income benefit or tax credit payment [44]. While there are some definitional changes over time (reflecting changes in the system of welfare benefits), the domain is broadly consistent. It has the added advantage of providing a score which is easy to interpret. Therefore, we use Income deprivation statistics for Scotland which were published for the years 2004, 2006, 2009 and 2012 at Datazone level in this study. Income Deprivation deciles in the study are defined separately for each year. 

Annual mean levels of air pollutants are not currently published for Datazones. Instead estimates of levels of PM_10_, PM_2.5_, NOx, NO_2_, SO_2_ and ozone are produced for points on a 1 km by 1 km square grid for the whole of the UK. To produce estimates for Datazones, the pollution grids are first overlaid with Datazone boundaries to calculate the areas of grid cells falling into a Datazone, and then the pollution level of that Datazone is calculated as the weighted averages of grid cell values (see Supplementary Material for more information). As the data available at 1 km × 1 km resolution are themselves modeled estimates, interpolated and extrapolated from data from several pollution monitoring stations by the Department for Environment Food & Rural Affairs [45] in the UK, the mean annual pollution estimates are in effect modeled twice. However, these data are to our knowledge still the best available data on local air pollution levels for the UK, and were produced to a standard that allows the UK to meet European air quality monitoring objectives, such as those specified in the 2008 EU Air Quality Directive (2008/50/EC), and the related Air Quality Framework directives (2004/107/EC and 1996/62/EC). City regions were defined using the Scottish Government’s Travel-to-Work Areas (TTWAs). These are functional geographies based on commuting flows, measured by the Census 2001. In the analyses below, we use PM_2.5_ as the measure of air pollution, but the findings in terms of the spatiotemporal nonlinearities in the relationship between air pollution and deprivation apply to other pollutants such as NO_2_ and SO_2_. Summary statistics on air pollution and deprivation in the study area are provided in Table 1.

PM_2.5_ concentrations in Scotland are not high on average, but there appears to be an increasing trend since 2009 (Table 1). As the spatial scales at which different theoretical processes underlying the link between deprivation and pollution discussed above operate are likely to be varying, we resort to the fine-grained spatial units Datazones in the whole of Scotland as our primary analysis units. The national-scale analysis with fine-resolution geographies produces opportunities of finding interesting, diverse and statistically reliable functional forms of linkage between deprivation and pollution due to large sample size. 

## 3. Results and Discussions

### 3.1. Explore Relationships without Imposing Functional Form

**Figure 1:**
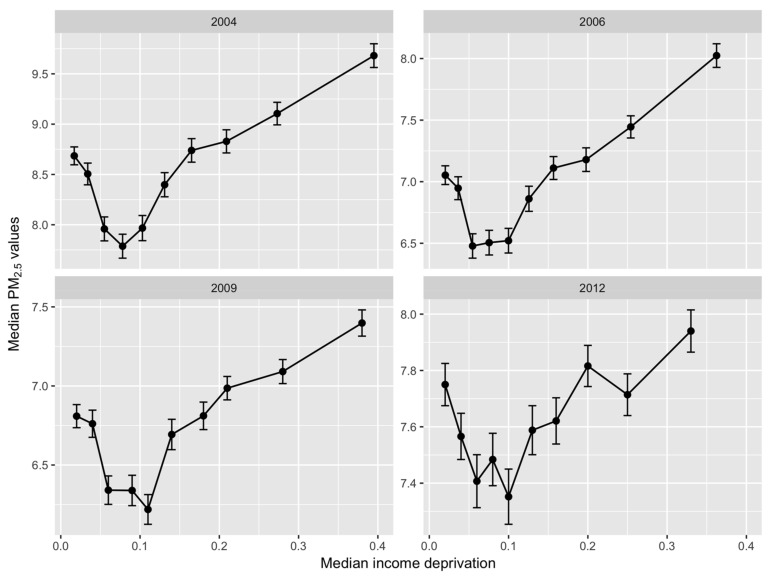
Pollution–deprivation relationship for Scotland during 2004–2012. Note: Median Income Deprivation is the median of scores in each decile.

Figure 1 depicts the median pollution levels for each decile of Income Deprivation across Scotland for the years 2004, 2006, 2009 and 2012. The panels show a similar non-linear, “tick” shaped relationship in all four years—a relationship also evident in [5,13] using data for Great Britain. Air pollution is highest in the most deprived deciles and falls steadily, reaching a minimum around the third to fifth deciles. In the least deprived two deciles, however, pollution begins to rise again although it remains lower than in the most deprived areas on average. 

**Figure 2:**
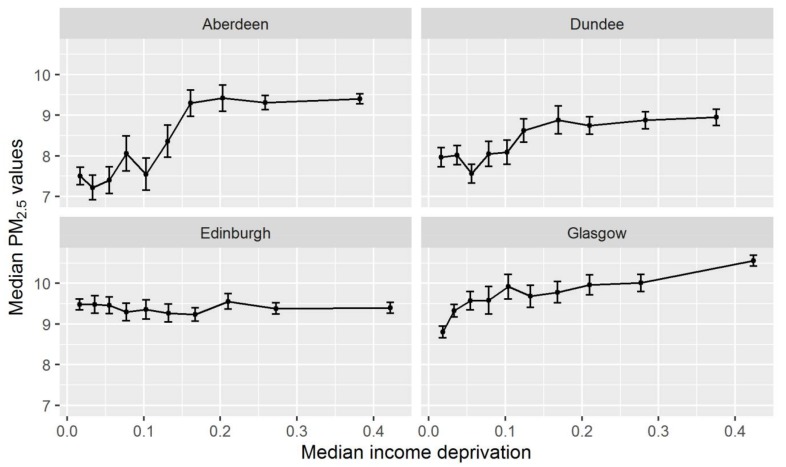
Pollution–deprivation relationship for four city-regions in 2004. Note: Deprivation deciles and associated median scores are defined at the national scale to facilitate comparison. These charts are plotted for 2004. Plots for 2006, 2009 and 2012 are also available as Supplementary Materials, available on request. Note: Median Income Deprivation is the median of scores in each decile.

Such a relationship may however be in part an artifact of the uneven geographical distribution of Income Deprivation between cities, combined with differences in levels of pollution between them. To explore the relationship locally, we impose pre-existing TTWA boundaries. For simplicity, we look just at the four largest TTWAs which account for around 45% of the total population. Figure 2 shows relationships in 2004. What is most noticeable here are the variations in the relationship between the cities and the fact that none of the cities has a pattern which resembles the national picture in Figure 1. Aberdeen and Dundee show some similarities with positive, largely monotonic relationships. Pollution is highest in the four or five most deprived deciles, then sharply lower in the least deprived. However, the variation in levels is far greater in Aberdeen. Glasgow also shows a positive, monotonic relationship but with a more gradual, continuous rise, with a range closer to Dundee’s. In Edinburgh, however, the relationship is essentially flat. Looking at the other years, there is some variation in the pollution–deprivation relationships over time at the city level but none corresponds to the Scottish picture (Figures S3–S5 in the Supplementary Materials). 

Regression models are employed to summarize the relationships, with Income Deprivation centered and air pollution transformed using a natural logarithm to reduce potential influence of heteroscedasticity on model estimation. Table 2 shows estimation results for Scotland for all four years and for the four cities for the first and last years. These results allow comparison with the results of the GWR models discussed in the next section. In the Scottish models, the coefficients for both Income Deprivation and its squared term are positive, recovering the “tick” shapes found in Figure 1. The models are broadly stable over time but decline in explanatory power, suggesting some change in the pollution–deprivation relationship. For the cities, the summaries are quite different. For Glasgow, Dundee and Aberdeen, the linear terms have a positive coefficient but the quadratic terms are negative. All three show a reduction in explanatory power over time. The models for Edinburgh have no explanatory value whatsoever at either period (R squared 0.001), reflecting the largely flat line in Figure 2. 

### 3.2. Local Spatial Non-Linear Analysis

The variations between the four cities prompt us to try to capture the relationships in a more sophisticated way without imposing an essentially arbitrary geography. Our theoretical discussion above suggests differences may emerge from a range of sources, reflecting current and historic city or local authority boundaries, and these will not necessarily be coterminous with current TTWA boundaries. A more flexible approach would be to allow the regression coefficients to vary across space continuously which is exactly what the GWR models are designed for [41]. 

GWR model results for 2004 and 2012 are summarized in Table 3. This approach produces a noticeable increase in the model fit; for 2004 and 2012, respectively, adjusted R-squared rises from 0.085 and 0.022 in the global model, to 0.827 and 0.791 with GWR. For a formal statistical test of GWR models against global regression models in both years, F-tests were conducted following [46] (Table 4). The results confirm that GWR models significantly outperform their counterpart global models, indicating a significant spatial variation in pollution–deprivation relationship across Scotland. A further question is whether the variations in local regression coefficients from the GWR models reflect complex processes linking Income Deprivation to air pollution, or are just driven by random noise in the data. To address this issue, a test of spatial non-stationarity proposed in [47] was conducted. The results are provided in the last column of Table 3 and clearly show that there is a systematic spatial patterning here. We also estimated GWR models for Glasgow in 2004 and 2012. The results are available in the Supplementary Materials, which also show systematic spatial patterns in the association between air pollution and deprivation.

**Figure 3:**
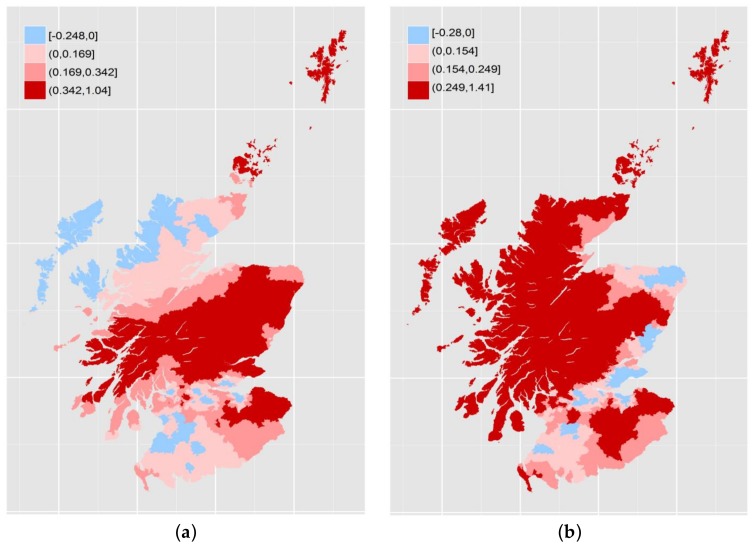
Local coefficients for income deprivation: 2004 (**a**); and 2012 (**b**).

**Figure 4:**
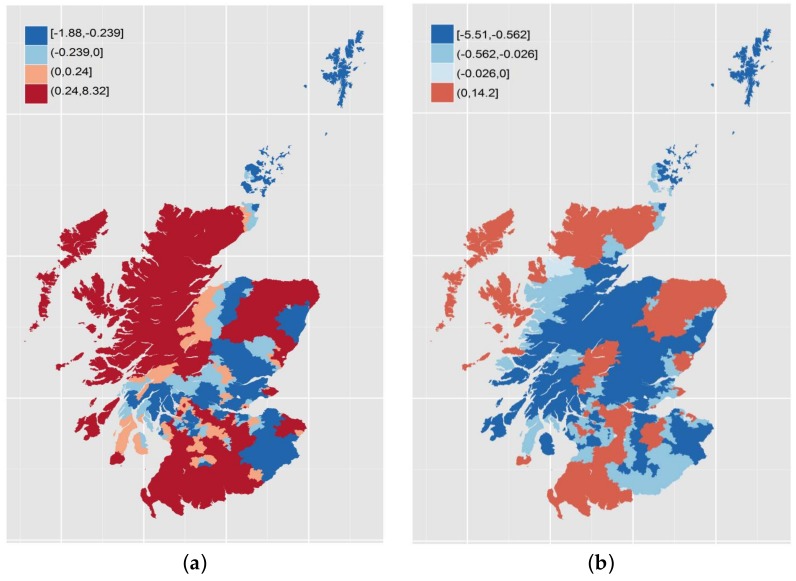
Local coefficients for squared income deprivation: 2004 (**a**); and 2012 (**b**).

Turning to the results themselves, we see that the median coefficient for the linear term is positive in both years while, for the quadratic term, it is negative in both cases—similar to the “global” models for Aberdeen, Dundee and Glasgow TTWAs. At the same time, there are locations with negative linear coefficients and positive quadratic terms. The geographic variations in linear and quadratic terms are shown in Figure 3 and Figure 4. The break points used in each case are zero, the median and the upper or lower quartile as appropriate to produce four bands. 

The results show a both variation over space but also change over time. The linear terms in Figure 3 show some degree of stability, particular in the distinction between the urbanized Central Belt of Scotland where the majority of the population lives and the more rural areas of the Highlands to the north and the Borders to the southeast. The southwest, covering Ayrshire, has more in common with the Central Belt with which it shares a similar industrial history. Over time, major change appears in the northwest and along the northeast coast. With the quadratic terms, there is less of an obvious geography and even greater change over time. There are also sharper spatial discontinuities. 

### 3.3. Identifying Regions

**Figure 5:**
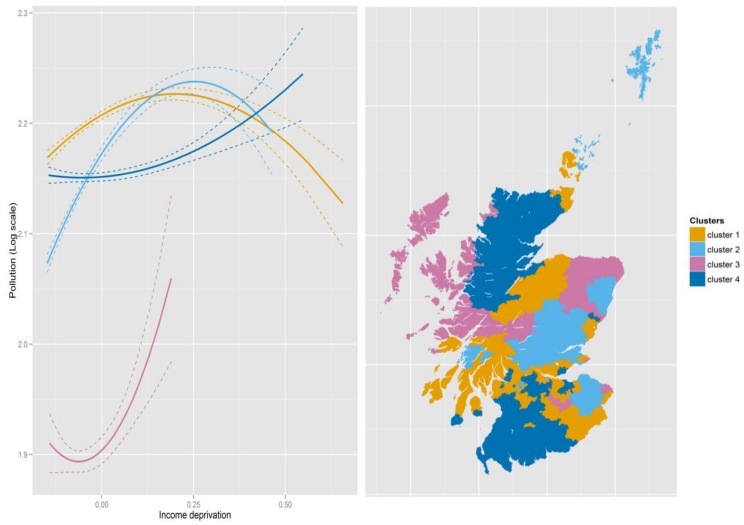
Pollution–deprivation relationships for the clusters in 2004. Note: The dashed lines are the approximate 95% confidence intervals.

**Figure 6:**
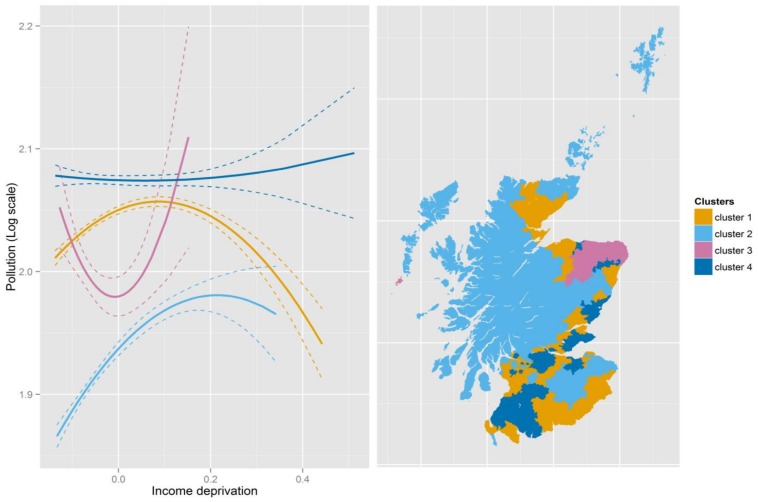
Pollution–deprivation relationships for the clusters in 2012. Note: The dashed lines are the approximate 95% confidence intervals.

Having identified significant spatial variations, our final step is to construct regions which share similar pollution–deprivation relationships. We use cluster analysis on the linear and quadratic terms. In 2004, four clusters are chosen; adding another only improves the explained total variations of the two variables marginally. For the purpose of comparison across years, we also choose four clusters in 2012. Summaries are provided in Table 5 while Figure 5 and Figure 6 depict the pollution–deprivation relationships for each cluster and the corresponding geographies of the clusters. Clusters are ordered so that each cluster has a similar composition in both years. Although we did not impose geographical proximity in our cluster algorithm, the identified clusters are reasonably compact in space. A similar observation was made by [43] where they identify crime areas by clustering GWR parameters. However, this can be expected as parameters estimated from GWR arise from models based on overlapping sets of areas. The approximate 95% confidence intervals for each curve are also shown in the figures. 

Cluster 3 is the smallest covering a relatively affluent set of areas where there is a “tick” shaped relationship similar to that for Scotland as a whole. The cluster does not cover the most deprived areas, however. In 2012, it is confined to more rural parts of the northeast of Scotland, while, in 2004, it covers the same area plus some additional rural areas in the highlands, islands and the borders. Cluster 1 is the largest (in population terms) in each case, covering regions where the relationship forms an inverted U, so pollution tends to be lower in the most deprived locations and highest in those just above average. There is similarity with Cluster 2 although that cluster does not cover as many deprived Datazones and tends to show greater improvement in the least deprived areas. Cluster 4 is the one which corresponds most clearly to the environmental justice expectations, with pollution rising steadily with deprivation, albeit not at a dramatic rate. 

## 4. Conclusions

When considered through a broad theoretical framework, we should expect the relationship between pollution and deprivation to be complex, varying both spatially and temporally. Cognizant of these theoretical expectations, we sought to deploy a flexible approach to empirical modeling, able to capture potential non-linearity, non-monotonicity, spatial variation and temporal fluidity in the relationship between pollution and deprivation. When we applied our approach to data for Scotland, we found both nonlinearity and spatiotemporal variability in the relationship. This leads to two rather unsettling conclusions. First, an implication of spatially varying relationships is that there is the potential for geographical selection bias—what researchers find may depend on where they choose to study the phenomenon. Second, using empirical methods that assume linearity, most obviously if calculating and presenting a linear trend coefficient, may lead to highly misleading results. 

One implication for policy makers of our findings is that that the issue of environmental injustice, particularly with respect to air pollution, needs to be understood at a local level. Though national and global targets for air pollution are important for ensuring no populations are exposed to particularly high levels of harmful pollutants, the relative distribution of air pollution exposure by deprivation can vary hugely between and within regions for reasons that cannot be attributed entirely to issues of environmental injustice. A second potential policy implication is that a positive relationship between air pollution and deprivation is not an inevitability, as the relationship in Edinburgh makes clear—the fact that the relationship appears to shift over time and space might suggest that it is amenable to policy intervention. 

Note that the empirical section of this work has not attempted to test for particular avenues of causation or intervention. There are therefore rich opportunities for future work to deepen our understanding of the relationship between particular theoretical causes and particular outcomes for the relationship between pollution and deprivation. For example, qualitative and case study research focused on particular communities in our study area could provide a valuable and important insights into the lived experience and local context of our findings. There are also considerable opportunities for methods innovation; for example, the cluster modeling approach presented here could be improved by the inclusion of algorithms that yield contiguous spatial clusters. This may help generate more stable local clusters for the pollution–deprivation relationship. Another fruitful avenue would be to decompose air pollution into its constituent sources and estimate separate relationships with deprivation for each source of emissions. This would help provide policy makers with a more nuanced picture of the social justice implications of pollution, such as whether those who emit the most of a particular type of pollution are also the ones most likely to be exposed to it.

## Figures and Tables

**Table 1:** Summary statistics for Income Deprivation and air pollution (PM_2.5_).

**Income Deprivation**	**Mean**	**Std. Dev**	**Lower Quantile**	**Upper Quantile**
2004	0.149	0.121	0.055	0.209
2006	0.141	0.110	0.055	0.198
2009	0.154	0.113	0.060	0.210
2012	0.138	0.098	0.060	0.200
**Air Pollution (PM2.5 Micrograms/m3 (μg m−3))**	**Mean**	**Std. Dev**	**Lower Quantile**	**Upper Quantile**
National				
2004	8.630	1.542	7.422	9.607
2006	7.088	1.284	6.205	7.937
2009	6.779	1.135	5.995	7.523
2012	7.557	1.087	6.888	8.236
Aberdeen				
2004	8.247	1.308	7.092	9.363
2006	6.523	1.024	5.630	7.338
2009	6.924	1.158	6.078	7.797
2012	8.169	0.887	7.677	8.665
Dundee				
2004	8.404	0.750	7.785	8.981
2006	7.455	1.233	6.264	8.328
2009	6.680	0.622	6.122	7.059
2012	7.609	0.448	7.325	7.921
Edinburgh				
2004	9.442	0.865	8.952	9.935
2006	7.716	0.765	7.122	8.271
2009	7.508	0.813	7.100	7.962
2012	8.497	0.701	8.130	8.874
Glasgow				
2004	9.986	1.357	8.967	10.878
2006	8.321	1.069	7.507	8.946
2009	7.602	0.947	6.965	8.057
2012	8.066	0.954	7.427	8.552

Note: We also derived estimates for other air pollutant levels, and summary statistics are available upon request.

**Table 2:** Estimation results from second-order polynomial regression models for Scotland and four city-regions during 2004–2012.

Variables	National Scale				Aberdeen		Dundee		Glasgow		Edinburgh	
	2004	2006	2009	2012	2004	2012	2004	2012	2004	2012	2004	2012
	Coefficients/(Std. Err)				Coefficients/(Std. Err)		Coefficients/(Std. Err)		Coefficients/(Std. Err)		Coefficients/(Std. Err)	
Intercept	2.132 *	1.936 *	1.892 *	2.006 *	2.157 *	2.118 *	2.133 *	2.027 *	2.283 *	2.079 *	2.243 *	2.139 *
	(−0.003)	(−0.003)	(−0.003)	(−0.002)	(−0.011)	(−0.009)	(−0.006)	(−0.005)	(−0.004)	(−0.004)	(−0.005)	(−0.004)
Income deprivation	0.358 *	0.448 *	0.261 *	0.164 *	0.74 *	0.357 *	0.466 *	0.113	0.433 *	0.374 *	0.043	0.011
	(−0.024)	(−0.026)	(−0.024)	(−0.024)	(−0.084)	(−0.079)	(−0.051)	(−0.047)	(−0.035)	(−0.037)	(−0.038)	(−0.038)
Squared income deprivation	0.453 *	0.435 *	0.548 *	0.554 *	−1.159	−0.128	−0.988 *	−0.139	−0.586 *	−0.788 *	−0.058	−0.246
	(−0.105)	(−0.126)	(−0.119)	(0.151)	(−0.588)	(−0.886)	(−0.269)	(−0.308)	(−0.12)	(−0.184)	(−0.176)	(−0.332)
Adjusted R^2^	0.085	0.092	0.05	0.022	0.14	0.043	0.265	0.026	0.139	0.083	0.001	0.001
Sample size	6505	6505	6505	6505	461	461	265	265	1398	1398	772	772

Note. The symbol “*” represents the significance level at 1%.

**Table 3:** GWR estimation results for 2004 and 2012.

Variables	Minimum	Lower Quartile (25%)	Median (50%)	Upper Quartile (75%)	Maximum	Non-Stationarity Test (F Statistic)
2004						
Intercept	1.701	2.065	2.192	2.282	2.504	516.3 *
Income deprivation	−0.248	0.039	0.169	0.342	1.044	11.81 *
Squared Income Deprivation	−1.884	−0.721	−0.239	0.240	8.324	5.87 *
Adjusted R^2^	0.827					
Residual standard error	0.075					
2012						
Intercept	1.663	1.968	2.047	2.123	2.262	383 *
Income deprivation	−0.28	0.020	0.154	0.249	1.409	7.52 *
Squared Income Deprivation	−5.508	−1.078	−0.562	−0.026	14.25	5.49 *
Adjusted R^2^	0.791					
Residual standard error	0.068					

Note: The symbol “*” represent significance level at 1%.

**Table 4:** ANOVA comparisons between GWR and global regression models.

Models	RSS	DF	MS	F	*p*
2004					
OLS (2004)	194.5	6502	0.03		
GWR	35.8	6336	0.006		
GWR improvement	158.7	165.7	0.958	169.7	0.000
2012					
OLS (2012)	141.2	6502	0.022		
GWR	29.4	6332	0.005		
GWR improvement	111.8	169.8	0.659	142.1	0.000

Note: RSS, residual sum of squares; DF, degrees of freedom; MS, mean squared error; F, F statistic values; *p*, associated *p* values associated with F statistics. OLS, the ordinal least squares model; GWR, geographically weighted regression model.

**Table 5:** Descriptive summaries for each cluster in 2004 and 2012.

Clusters	Cluster Summaries			Income Deprivation Summaries		
	Income Deprivation	Squared Income Deprivation	Cluster Size	Median	Min	Max
2004	Coefficients					
cluster 1	0.19	−0.48	2486	0.12	−0.15	0.66
cluster 2	0.52	−1.02	1377	0.08	−0.15	0.46
cluster 3	0.33	2.61	308	0.10	−0.14	0.19
cluster 4	0.03	0.26	2334	0.14	−0.15	0.55
2012	Coefficients					
cluster 1	0.16	−0.92	3008	0.12	−0.14	0.44
cluster 2	0.41	−0.95	1324	0.08	−0.13	0.34
cluster 3	0.08	5.08	164	0.09	−0.13	0.15
cluster 4	−0.01	0.11	2009	0.14	−0.14	0.51

## Supplementary Materials

The following are available online at http://www.mdpi.com/1660-4601/15/4/629/s1. Figure S1: The PM_2.5_ values at 1 km × 1 km grid cells from the Defra (left) and estimated values for Datazones in 2004, Figure S2: The PM_2.5_ values at 1 km × 1 km grid cells from the Defra (left) and estimated values for Datazones in 2012, Figure S3: Pollution–deprivation relationship for four city-regions in 2006, Figure S4: Pollution–deprivation relationship for four city-regions in 2009, Figure S5: Pollution–deprivation relationship for four city-regions in 2012, Figure S6: Local coefficients for income deprivation (a) and squared income deprivation (b) in Glasgow in 2004, Figure S7: Local coefficients for income deprivation (a) and squared income deprivation (b) in Glasgow in 2012, Table S1: GWR estimation results for Glasgow TTWA—2004 and 2012.
