# Supplementary material for: Reconsidering the Relationship between Air Pollution and Deprivation
Source: Int J Environ Res Public Health. 2018 Mar 29;15(4):629. doi: 10.3390/ijerph15040629 (PMC5923671; doi:10.3390/ijerph15040629)
Supplement: Supplementary file 1 [file ijerph-15-00629-s001.pdf]

Supplementary Material

A brief illustration of the GWR model

In most of GWR applications, an optimum bandwidth that maximises the out-of-sample model fit (or a cross-validation procedure) is used. We decided not to do so in the present study. First, the bandwidth obtained through an optimum procedure is less than 20, which is about 0.3% of the sample size. With this bandwidth, we observe a lot of extreme regression coefficients. More importantly, there is a serious issue of multi-collinearity in local regression coefficients from a GWR model with a bandwidth calibrated by using the cross-validation procedure, which might seriously affect model estimates (Wheeler and Tiefelsdorf, 2005; Páez et al., 2011).

GWR model results presented in the present study is based on an adaptive kernel with a bandwidth of 400 (about 6% of the total number of Datazones) where we found no evidence of multi-collinearity as all the local condition numbers (the ratio of the largest to the smallest singular values of the model design matrix at each location) are less than 22, below the conventional threshold for concern of 30 (Fotheringham et al. 2002). We also used 4% and 10% of the total sample as bandwidths to run GWR models and the results were very similar to those reported in the main text.

Other approaches are also available for an exploration of local associations between air pollution and income deprivation such as hierarchical Bayesian spatially varying coefficients models (SVC, Assuncao, 2003; Gelfand et al., 2003). Unlike the GWR model, SVC is a global model with the variations of coefficients specified by either a geostatistical process (Gelfand et al., 2003) or a multivariate conditional autoregressive process (Assuncao, 2003). These sophisticated models are less subject to issues of multi-collinearity and multiple hypotheses testing that can sometimes plague the estimation and statistical inference of GWR. Nonetheless, SVC models are extremely computing intensive and can be practically impossible to be applied to large spatial data sets.

Mapping grid cell level pollution measures to Datazones

Annual mean levels of air pollutant levels are not available at the Datazone level. Instead, modelled background air pollution concentrations (e.g. PM10, PM2.5, NOX, NO2, SO2 and Ozone) are produced for a series of 1km by 1km square grid cells for the whole of the UK (http://uk-air.defra.gov.uk/). We use these grid estimates to interpolate pollution summaries for each Datazone in Scotland. For the majority of Datazones, there are several grid cells falling into a Datazone. In which case, the weighted averages of grid estimates are used to represent the pollution level of a Datazone with weights being the areas of grid cells falling into it divided by the total area of that Datazone. For example, if a Datazone (with an area of 2.5 km2) consists of three grid cells with two cells completely falling into it and one only a half, the weights assigned to these three grid cells are 0.4, 0.4 and 0.2, respectively. In cases where a Datazone was completely falling into a grid cell (for example in highly-populated urban areas) or a Datazone was only intersected with one single grid cell, the grid pollution estimate was assigned to that Datazone. The estimated datazone pollution measures quite nicely replicate the features and spatial pattern of the original grid cell pollution summaries (see Figures S1 and S2).

Additional plots for the pollution-deprivation relationship for four city-regions

In addition to the chart plotted for 2004 included in the main document (Figure 2), we also present plots below for 2006, 2009 and 2012 (see Figure S3, Figure S4 and Figure S5 respectively). Deprivation deciles and associated median scores are defined at the national scale to facilitate comparison.

GWR model estimation results for Glasgow TTWA

The GWR estimation results on the spatial variability in the association between air pollution and deprivation for Glasgow are provided in Table S1. They are obtained by using an adaptive GWR model with a bandwidth of 84 (about 6% of the total number of Datazones in Glasgow TTWA). In line with the findings from the national-scale analysis in the main text, there is statistically significant variability in the association between air pollution and deprivation, as indicated by the non-stationarity test results. The spatial patterns in the associations between air pollution and the linear and quadratic income deprivation terms in 2004 and 2012 are shown in Figures S6 and S7.

Table S1. GWR estimation results for Glasgow TTWA - 2004 and 2012.

	**Minimum**	**Lower Quartile (25%)**	**Median (50%)**	**Upper Quartile (75%)**	**Maximum**	**Non-Stationarity Test (F Statistic)**
2004						
Intercept	2.052	2.205	2.304	2.383	2.612	267.9 *
Income deprivation	-0.714	-0.036	0.078	0.191	0.575	8.662 *
Squared Income Deprivation	-2.890	-0.449	-0.101	0.151	3.031	4.315*
Adjusted R^2	0.934					
2012						
Intercept	1.87	2.018	2.08	2.142	2.344	141.2 *
Income deprivation	-1.001	-0.064	0.083	0.204	0.724	6.29 *
Squared Income Deprivation	-2.546	-0.713	-0.111	0.459	6.188	3.742*
Adjusted R^2	0.874					

Note: The symbol "*" represent significance level at 1%.

Figures

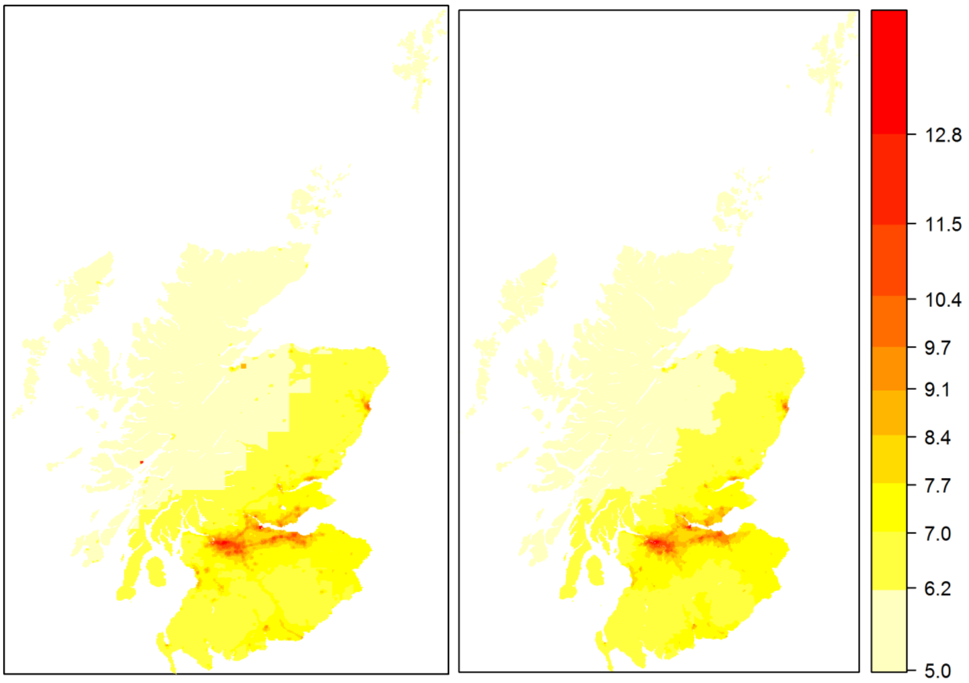

Figure S1. The $PM_{2.5}$ values at 1 km by 1 km grid cells from the Defra (left) and estimated values for Datazones in 2004/

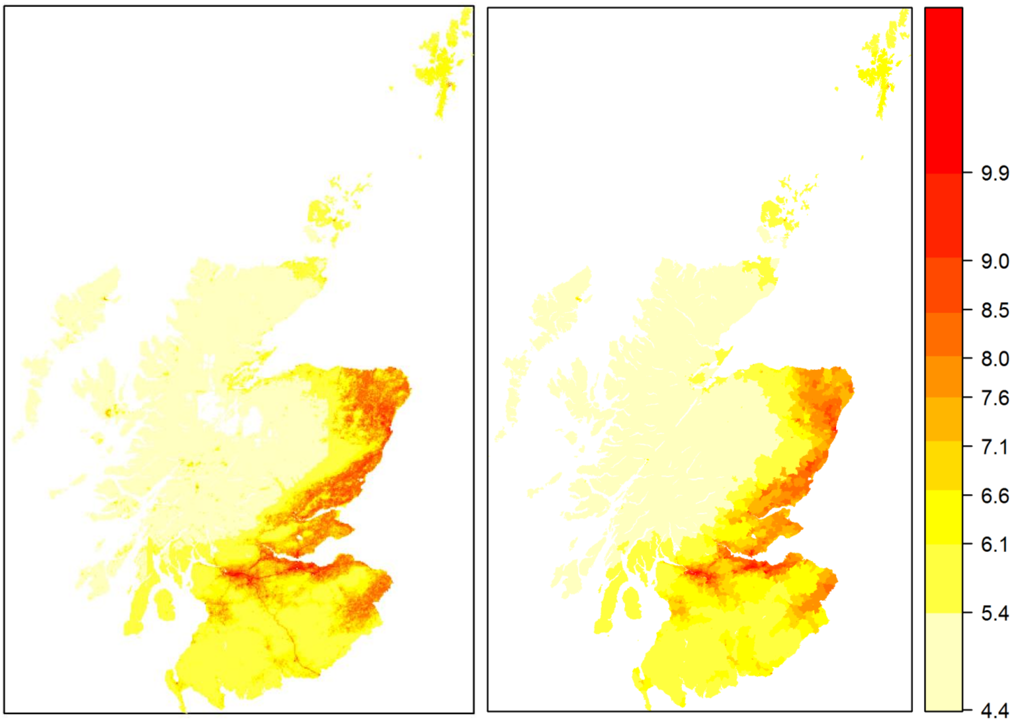

Figure S2. The $PM_{2.5}$ values at 1 km by 1 km grid cells from the Defra (left) and estimated values for Datazones in 2012.

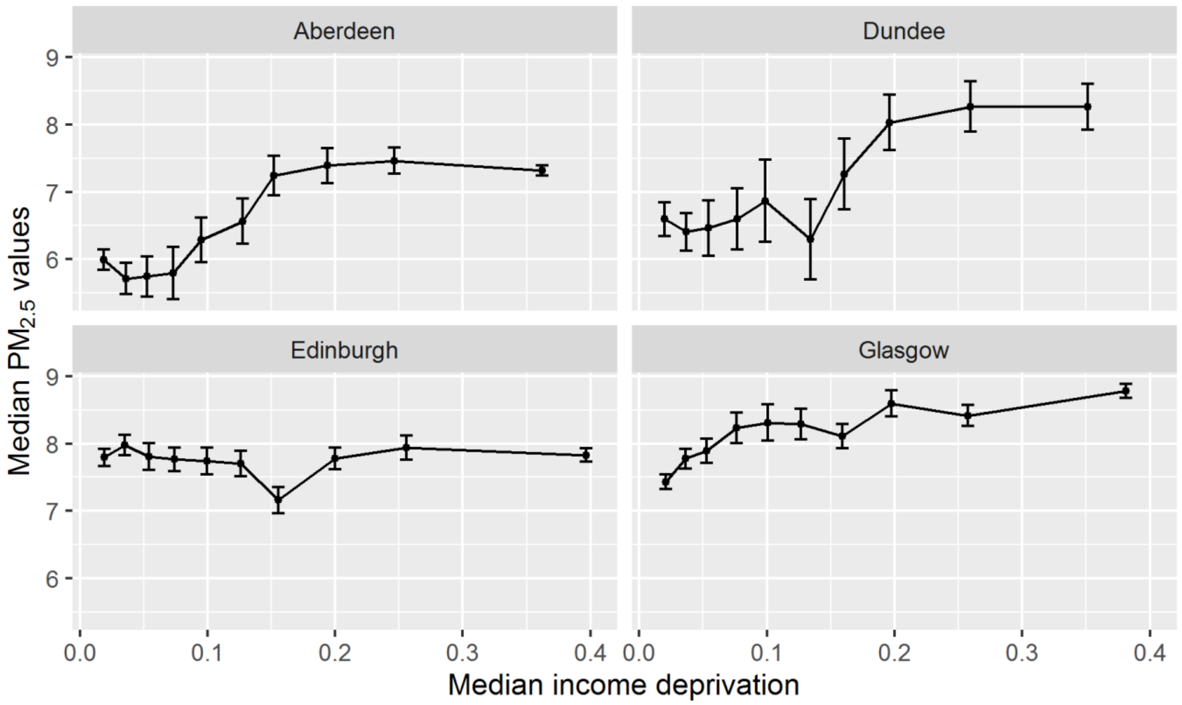

Figure S3. Pollution-deprivation relationship for four city-regions in 2006.

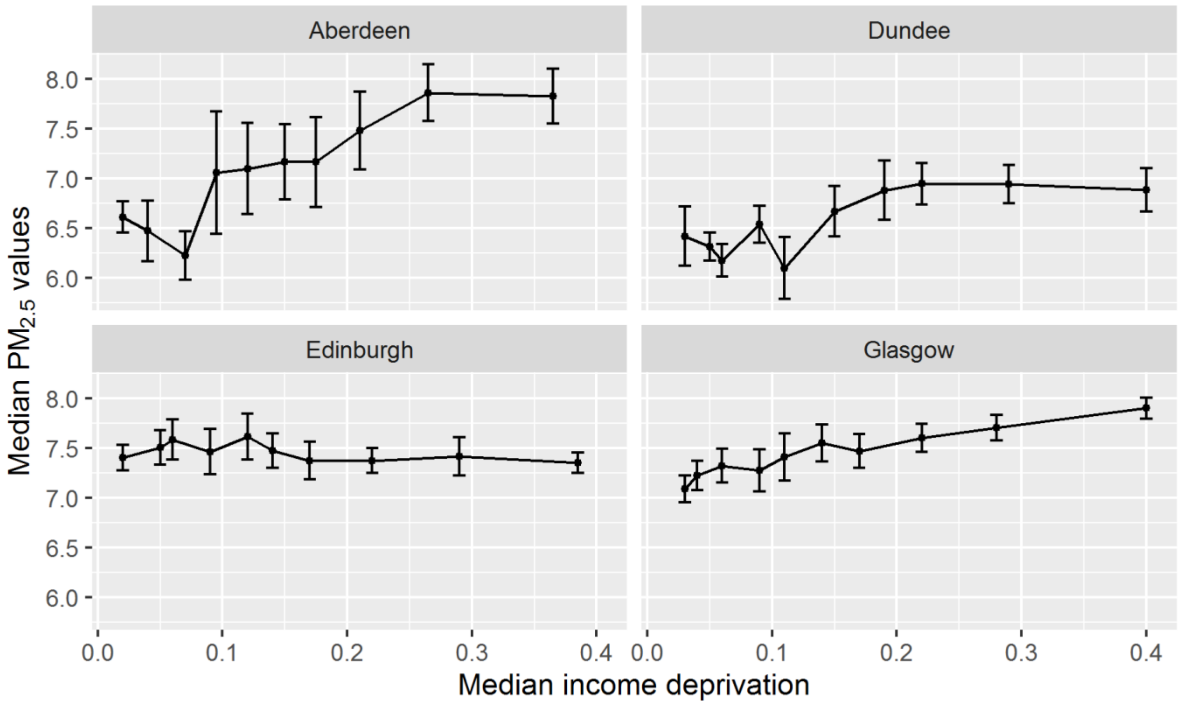

Figure S4. Pollution-deprivation relationship for four city-regions in 2009.

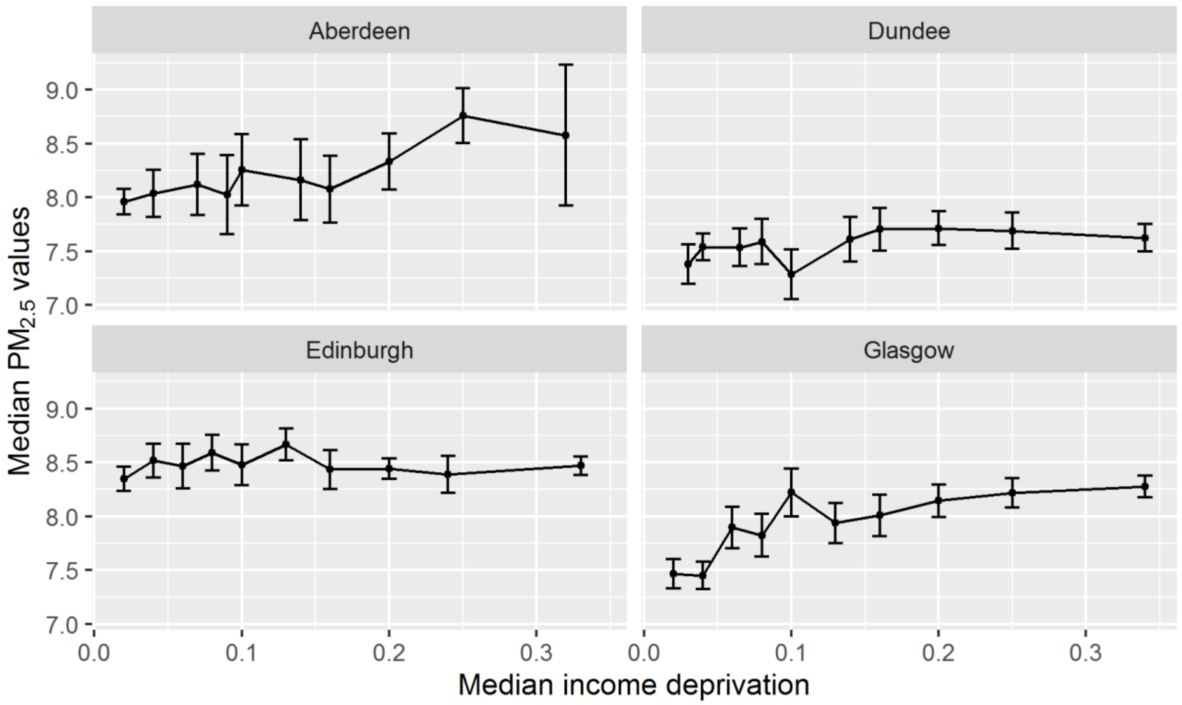

Figure S5. Pollution-deprivation relationship for four city-regions in 2012.

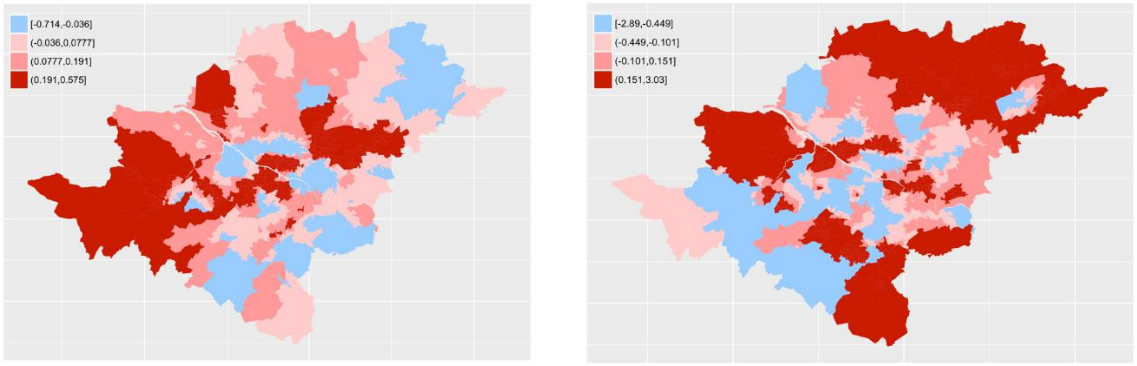

(**a**) (**b**)

Figure S6. Local coefficients for income deprivation (**a**) and squared income deprivation (**b**) in Glasgow in 2004.

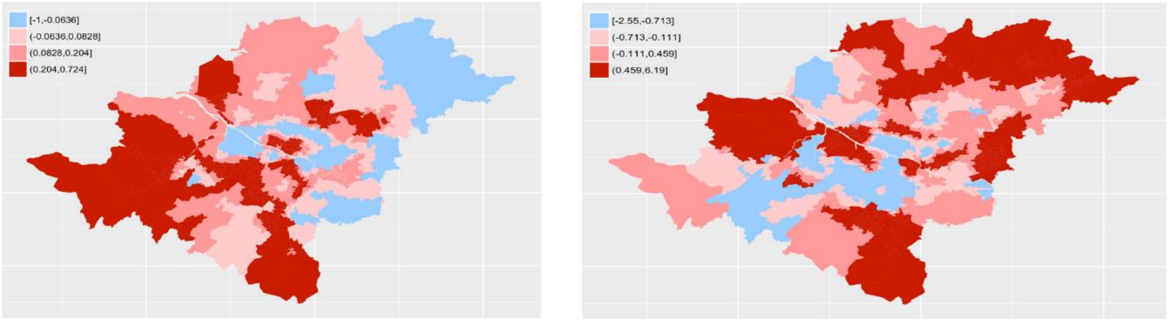

(**a**) (**b**)

Figure S7. Local coefficients for income deprivation (**a**) and squared income deprivation (**b**) in Glasgow in 2012.

References

1. Assunção, R.M. (2003). Space varying coefficient models for small area data. *Environmetrics*, 14, 453–473.
2. Fotheringham A.S, Brunsdon C, and Charlton M (2002). *Geographically Weighted Regression: The Analysis of Spatial Varying Relationships*. Chichester, John Wiley and Sons
3. Gelfand A.E, Kim H, Sirmans C.F, and Banerjee S. (2003). Spatial modelling with spatially varying coefficient processes. *Journal of the American Statistical Association*, 98: 387–396.
4. Páez A.; Farber S.; Wheeler D. (2011). A simulation-based study of geographically weighted regression as a method for investigating spatially varying relationships. *Environment and Planning A* 43: 2992–3010.
5. Wheeler D. and Tiefelsdorf M. (2005). Multicollinearity and correlation among local regression coefficients in geographically weighted regression. *Journal of Geographical Systems*, 7: 161–187.
